# Protein Deficiency-Induced Behavioral Abnormalities and Neurotransmitter Loss in Aged Mice Are Ameliorated by Essential Amino Acids

**DOI:** 10.3389/fnut.2020.00023

**Published:** 2020-03-11

**Authors:** Hideaki Sato, Masako Tsukamoto-Yasui, Yuhei Takado, Noriko Kawasaki, Keiko Matsunaga, Satoko Ueno, Mayuka Kanda, Mai Nishimura, Sachise Karakawa, Muneki Isokawa, Katsuya Suzuki, Kenji Nagao, Makoto Higuchi, Akihiko Kitamura

**Affiliations:** ^1^Ajinomoto Co., Inc., Kawasaki, Japan; ^2^Department of Functional Brain Imaging, National Institute of Radiological Sciences, National Institutes for Quantum and Radiological Sciences and Technology, Chiba, Japan

**Keywords:** protein deficiency, low protein diet, essential amino acids, cognitive function, neurotransmitter, aged mouse, passive avoidance test, elevated plus maze test

## Abstract

Nutritional epidemiology shows that insufficient protein intake is related to senile dementia. The levels of protein intake in aged people are positively associated with memory function, and elderly people with high protein intake have a low risk of mild cognitive impairment. Although the beneficial roles of protein nutrition in maintaining brain function in aged people are well demonstrated, little is known about the mechanism by which dietary intake of protein affects memory and brain conditions. We fed aged mice a low protein diet (LPD) for 2 months, which caused behavioral abnormalities, and examined the nutritional effect of essential amino acid administration under LPD conditions. The passive avoidance test revealed that LPD mice demonstrated learning and memory impairment. Similarly, the LPD mice showed agitation and hyperactive behavior in the elevated plus maze test. Moreover, LPD mice exhibited decreased concentrations of gamma-aminobutyric acid (GABA), glutamate, glycine, dopamine, norepinephrine, serotonin and aspartate in the brain. Interestingly, oral administration of seven essential amino acids (EAAs; valine, leucine, isoleucine, lysine, phenylalanine, histidine, and tryptophan) to LPD mice, which can be a source of neurotransmitters, reversed those behavioral changes. The oral administration of EAAs restored the brain concentration of glutamate, which is involved in learning and memory ability and may be associated with the observed behavioral changes. Although the details of the link between decreased amino acid and neurotransmitter concentrations and behavioral abnormalities must be examined in future studies, these findings suggest the importance of dietary protein and essential amino acids for maintaining brain function.

## Introduction

Alzheimer's disease (AD) is a neurodegenerative condition that is highly prevalent in old age (1) and has a significant socioeconomic impact, which will lead to an increased economic burden in healthcare systems worldwide (2). Because pathological changes, such as amyloid β accumulation, occur more than two decades before the appearance of cognitive impairments (1), finding preventive strategies against AD is important. However, prescribing drugs for AD prevention to people several decades before the onset of cognitive impairments has huge socioeconomic impact considering the growing number of patients with AD (3). In this context, daily food intervention could be a realistic strategy for AD prevention.

Indeed, nutritional epidemiology has shown the importance of protein intake for maintaining brain function in the elderly population. Compared with the healthy elderly, patients with dementia have significantly lower protein intake and lower protein intake of patients with dementia is reported to be associated with severe dementia (4–6). The levels of protein intake in aged people are positively associated with memory function (7, 8), and elderly people with high protein intake have a low risk of mild cognitive impairment (MCI) (9). Moreover, elderly people with high protein intake have recently been reported to have low amyloid β accumulation in the brain (10).

Although the beneficial roles of protein nutrition for brain function in aged people are well demonstrated, little is known about the mechanism by which protein intake maintains brain function and prevents MCI. Given that proteins are composed of multiple amino acids, including essential amino acids (EAAs), protein malnutrition could lead to amino acid intake deficiency, thereby affecting the brain. Amino acids are known to play essential roles not only as energy sources but also in protein synthesis, metabolism and homeostatic function of cells in multiple organs of the body, including the brain. Amino acids function as precursors of neurotransmitters, especially in the brain. We hypothesized that a low protein diet (LPD) leads to low concentrations of EAAs in the plasma and brain, resulting in a depletion of neurotransmitters in the brain. To unveil the nutritional importance of protein and amino acids in brain function, we fed aged mice a LPD, which caused behavioral abnormalities, and further examined the nutritional effect of seven EAAs [valine (Val), leucine (Leu), isoleucine (Ile), lysine (Lys), phenylalanine (Phe), histidine (His), and tryptophan (Trp)], which are sources of neurotransmitters, in this model.

## Materials and Methods

### Animals

Male C57BL/6J mice (55–63 weeks, Charles River Laboratories, Japan) were used for experiments. These mice were housed at 25°C on a 12-h light/dark cycle (lights on 8 PM to 8 AM) with *ad libitum* food and water in their cages. All animal experimental procedures in the present study were approved by the institutional review board of the animal ethical committee, who follows the institutional guidelines of Ajinomoto Co., Inc.

### Diet and Amino Acid Intervention

**Figure 1:**
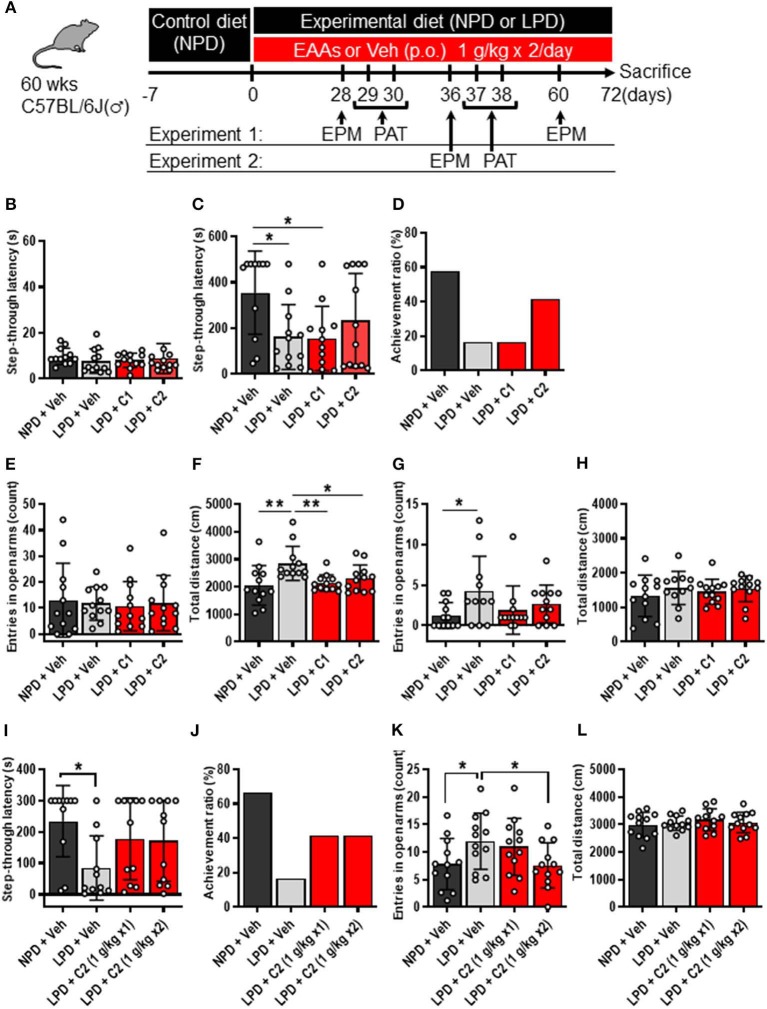
LPD induced cognitive decline, agitation, and disinhibition behavior, but EAAs ameliorated these changes. **(A)** Schematic diagram of the experimental procedure. Experiment 1 data are shown in **(B–H)**. **(B–D)** Summary of PAT results. **(B)** During the training session, there was no significant difference among the groups. **(C)** Mean step-through latency in each group during the training trial [F_(3, 43)_ = 3.4, *p* < 0.05]. The latency time was significantly lower in the LPD + Veh group than in the NPD + Veh group (*p < 0.05) and in the LPD + C1 group than in the NPD + Veh (*p < 0.05) but not in the LPD + C2 group compared with that in the NPD + Veh group. **(D)** Summary of the achievement ratio which is the effect of the 1st electrical stimulation (ES) on step-through latency in each group. **(E–H)** Summary of EPM test results on day 28 **(E,F)** and day 60 **(G,H)**. **(E)** Mean number of entries in the open arms on day 28 in each group. There was no significant difference among the groups. **(F)** Mean total distance traveled on day 28 in each group [F_(3, 44)_ = 5.0, *p* < 0.01]. The total distance was significantly increased in the LPD + Veh group compared with that in the NPD + Veh group (**p < 0.01, Holm-Sidak's test), in the LPD + Veh group compared with that in the LPD + C1 group (**p < 0.01, Holm-Sidak's test), and in the LPD + Veh group compared with that in the LPD + C2 group (*p < 0.05, Holm-Sidak's test). **(G)** Mean number of entries in the open arms on day 60 in each group (*p* < 0.05, Bartlett's test). The number of entries in the open arms was significantly higher in the LPD + Veh group than in the NPD + Veh group (*p < 0.05, Dunnett's test). **(H)** Mean total distance traveled on day 60 in each group. There was no significant difference among the groups. Experiment 2 data are shown in **(I–L)**. **(I,J)** Summary of PAT results. **(I)** Mean step-through latency in each group during the training trial [F_(3, 40)_ = 2.9, *p* < 0.05]. The latency time was significantly decreased in the LPD + Veh group compared with that in the NPD + Veh group (*p < 0.05). **(J)** Summary of the achievement ratio in each group. **(K,L)** Summary of EPM test results. **(K)** Mean number of entries in the open arms in each group [F_(3, 44)_ = 4.1, *p* < 0.05]. The number of entries in the open arms was significantly higher in the LPD + Veh group than in the NPD + Veh group (*p < 0.05) and in the LPD + C2 (1 g/kg x2) group than in the LPD + Veh group (*p < 0.05). **(L)** Mean total distance in each group. There was no significant difference among the groups. Error bars and dots indicate SD and scores of individual mice, respectively. NPD, normal protein diet; LPD, low protein diet; EPM, elevated plus maze; PAT, passive avoidance test; Veh, vehicle; p.o., per os; C1, composition 1; C2, composition 2.

The mice were provided *ad libitum* access to water and a control diet [normal protein diet (NPD); 20% casein-based diet, Supplementary Table 1]. At the start of the experimental protocol, the control diet was replaced with the experimental diet, which was either the NPD (20% casein-based diet) or LPD (5% casein-based diet) (Supplemental Table 1). In the amino acid intervention conditions, we treated mice with 10 ml/kg 0.5% methylcellulose [vehicle (Veh)] per os (PO), 1 g/10 ml/kg composition 1 (C1) amino acids PO, or 1 g/10 ml/kg C2 amino acids PO (Table 1) twice daily on days 1–5 of each week during the experimental protocol period of 72 days (Figure 1A).

**Table 1:** Composition of amino acids mixture.

	**C1 (%)**	**C2 (%)**
Leucine	15.3	31.1
Lysine	21.6	22.1
Valine	2.7	3.9
Isoleucine	2.7	8.6
Phenylalanine	16.9	28.3
Histidine	40.0	5.4
Tryptophan	0.7	0.7
Total	100.0	100.0

### Quantification of Amino Acid and Monoamine Concentrations in the Plasma and Brain Tissue

A previously described quantification method for amino acids (11) was used in this study with minor modifications. The plasma sample was mixed with the internal standard solution (stable isotope-labeled amino acids in water) and deproteinized with acetonitrile. Frozen brain tissue was powdered using a Multi-Beads Shocker (Yasui Kikai, Osaka, Japan) and homogenized in an ice-cold methanol aqueous solution containing L-phenyl-*d*_5_-alanine was used to calculate recovery of the pretreatment procedure. The homogenate was further mixed with water and chloroform, and its upper phase was dried up. The residual was dissolved with water, and mixed with the internal standard solution. The plasma and brain samples were derivatized with APDSTAG® (FUJIFILM Wako Pure Chemicals, Osaka, Japan) and analyzed using liquid chromatography coupled with tandem mass spectrometry (LC-MS/MS) as described in (11). Dopamine, norepinephrine and serotonin measurements were conducted on an HPLC-ECD system (HTEC-500: EICOM, Kyoto, Japan) and expressed as pg/mg tissue weight. Plasma albumin and total protein and glucose measurements were conducted on a chemical analyzer (DRI-CHEM3500V: FUJIFILM, Tokyo, Japan).

### Elevated Plus Maze

The elevated plus maze (EPM) consisted of two open (29.5 × 6 cm) and two closed arms (29.5 × 6 × 15 cm), which extended from a central platform (6 × 6 cm) at 50 cm from the ground (BRC, Nagaoya, Japan). Each individual mouse was placed in the center area facing an open arm and allowed to freely explore the maze for 8 min. The behavior of the animals was recorded, tracked, and analyzed with the SMART 3.0 video tracking systems. The following parameters were evaluated: number of entries into the closed vs. open arms, distance traveled (cm) within the closed and opened arms, and time spent in the closed and opened arms. The EPM test was conducted on day 28 and day 60 in Experiment 1 and on day 36 in Experiment 2 during the dark period.

### Passive Avoidance Test

The passive avoidance test (PAT) was performed using a step-through cage (Muromachi Kikai, Tokyo, Japan) consisting of white and black compartments separated by a sliding door. During the training trial, mice were placed in the white compartment, the door was opened, and the step-through latency was recorded. When the mice entered the dark compartment with its four paws on the grid floor, an electric foot shock (1 mA) was delivered through stainless-steel rods for 1 s. After 24 h, a probe test was performed using the same procedure without any foot shock. The step-through latency time to enter the dark compartment was recorded up to a maximum of 480 s in Experiment 1 and 300 s in Experiment 2 as the cut-off latency. The PAT was conducted on days 29 and 30 in Experiment 1 and on days 37 and 38 in Experiment 2 during the dark period.

### Statistics

Statistical analyses were performed using GraphPad Prism 6 Software. Data were statistically analyzed by Welch's *t*-test for ≤2 comparisons and one-way analysis of variance (ANOVA) with Tukey's or Dunnett's or Holm-Sidak's posttest for ≥3 comparisons. *P* values of ≤0.05 were considered statistically significant at a confidence interval of 95%.

## Results

First, we examined whether LPD intake affected brain function in aged mice through behavioral experiments. The PAT was used to investigate the learning and memory activities of mice. In the training trial of the PAT, there was no significant difference in step-through latency among the groups (Figure 1B), whereas in the test trial, step-through latency was significantly lower in the LPD + Veh group than in the NPD + Veh group (Figure 1C). In addition, the achievement ratio was lower in the LPD + Veh group than in the NPD + Veh group (Figure 1D). The EPM is considered to be a reliable indicator of anxiogenic behavior and depends upon the assumption that mice inherently prefer the closed arms of the maze to the open arms. We observed the number of entries in the open arms and total distance in the EPM, which are given in Figures 1E–H. The percent time spent in the open arms was not significantly different among the groups (Figure 1E), whereas the total distance traveled was significantly greater in the LPD + Veh group than in the NPD + Veh group on day 28 (Figure 1F). On day 60, the total distance traveled was not significantly different among the groups (Figure 1H), whereas the number of entries (Figure 1G) and the percent time spent [F_(3, 43)_ = 4.8, *p* < 0.01, Brown-Forsythe test; *p* < 0.05, data not shown] in the open arms were significantly increased in the LPD + Veh group compared with that in the NPD + Veh group. These results indicate that LPD induced cognitive function decline and agitation and disinhibition behavior in aged mice.

**Figure 2:**
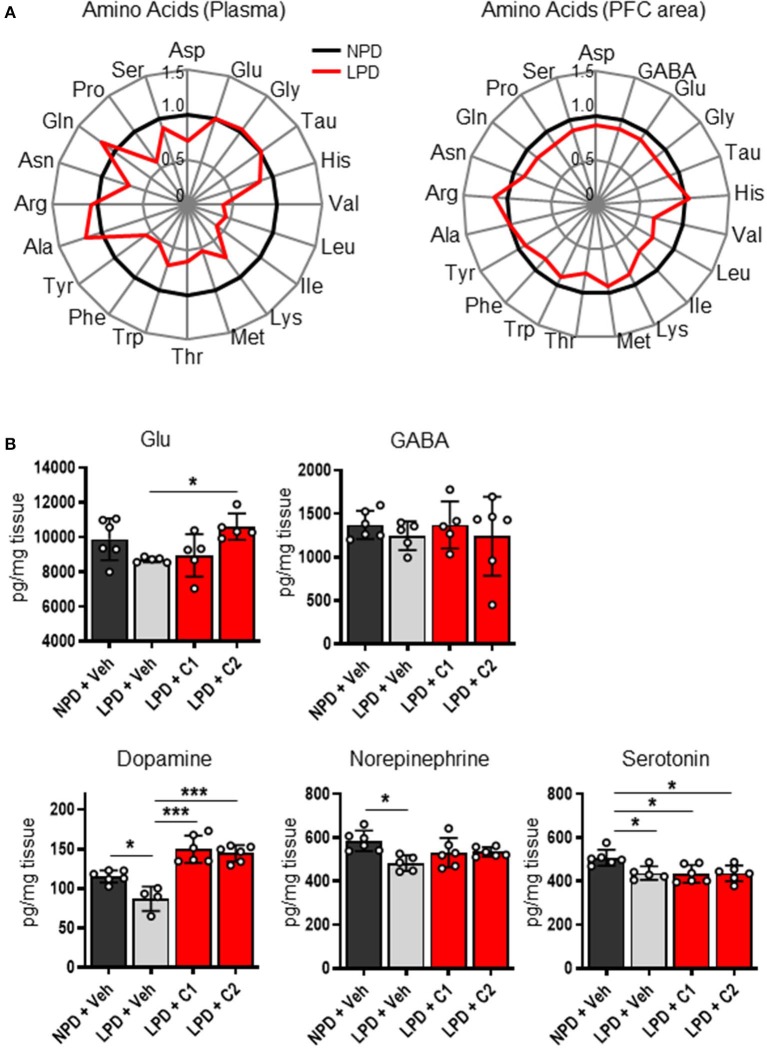
The concentrations of amino acids and neurotransmitters in the plasma and brain were decreased by LPD. **(A)** Radar charts of amino acids in aged B6 mice. The average of each amino acid concentration as normalized values in the plasma (left) and prefrontal cortex area (PFC area; right) are expressed. **(B)** Mean neurotransmitter concentrations in the PFC area after sacrifice in each group. The Glu concentration [F_(3, 17)_ = 4.2, *p* < 0.05] was significantly lower in the LPD + C2 group than in the LPD + Veh group (*p < 0.05). The dopamine concentration [F_(3, 18)_ = 23.9, *p* < 0.001] was significantly lower in the LPD + Veh group than in the NPD + Veh group (*p < 0.05), in the LPD + Veh group than in the LPD + C1 group (***p < 0.001), and in the LPD + Veh group than in the LPD + C2 group (***p < 0.001). The norepinephrine concentration [F_(3, 19)_ = 4.5, *p* < 0.05] was significantly lower in the LPD + Veh group than in the NPD + Veh group (*p < 0.05). The serotonin concentration [F_(3, 19)_ = 5.7, *p* < 0.01] was significantly lower in the LPD + Veh group than in the NPD + Veh group (*p < 0.05), in the LPD + C1 group than in the NPD + Veh group (*p < 0.05), and in the LPD + C2 group than in the NPD + Veh group (*p < 0.05). Error bars and dots indicate SD and scores of individual mice, respectively.

Next, we hypothesized that LPD leads to low concentrations of EAAs in the plasma and brain, thereby inducing neurotransmitter depletion in the brain, resulting in cognitive dysfunction and behavioral abnormalities. To identify the concentrations of EAAs and neurotransmitters, we quantified these concentrations in the plasma and brain of the NPD and LPD groups. Plasma levels of EAAs (Val, Leu, Ile, Lys, Met, Thr, Trp, and Phe) and non-essential amino acids (Tyr and Pro) in the LPD group were significantly lower than those in the NPD group (Figure 2A, Supplementary Table 2). The LPD group also exhibited significantly lower levels of EAAs (Val, Leu, Ile, Lys, and Thr), non-essential amino acids, and neurotransmitters [especially aspartate (Asp), GABA, glutamate (Glu), glycine (Gly), dopamine, norepinephrine, and serotonin] in the brain than the NPD group (Figure 2B, Supplementary Table 3). However, the concentrations of plasma albumin and total protein and glucose were not different between the LPD and NPD groups (Supplementary Figure 1).

Finally, we hypothesized that the flux of EAAs from the blood to the brain would be important for maintaining neurotransmitters. Thus, we conducted oral administration of seven EAAs (Val, Leu, Ile, Lys, Phe, His, and Trp), which are a source of neurotransmitters. To examine the importance of the flux of EAAs into the brain, we compared the effects of administering EAAs in the form of C1 (control against C2) or C2, which is composed of EAAs with high fluxes into the brain based on a previous report (12) (Table 1). Both C1 and C2 EAAs ameliorated the changes in agitation and disinhibition behavior indicated by a reversal of the changes in the total distance traveled on day 28 and the number of entries in the open arms on day 60 in the EPM (Figures 1F,G). In addition, C2 ameliorated the step-through latency in the PAT (Figure 1C), confirming the importance of fluxes of EAAs into brain for cognitive function. While C2 intake once per day was enough to ameliorate cognitive decline (Figures 1I,J), C2 intake twice per day was needed to ameliorate agitation and disinhibition behavior (Figure 1K). Moreover, C2 reversed the concentrations of Glu and dopamine in the brain (Figure 2B). However, there was no difference among the groups in the total distance in the EPM (Figure 1L).

## Discussion

Here, we demonstrated the importance of protein and amino acid nutrition for maintaining brain function. In this study, protein malnutrition in aged mice caused behavioral abnormalities as well as physiological alterations in the brain, including decreased neurotransmitter and plasma amino acid levels. These findings are in accordance with previous clinical studies showing the possibility that chronic protein malnutrition leads to cognitive dysfunction (7, 8). In this study, the changes induced by LPD were reversed by EAA supplementation, suggesting the importance of EAA nutrition in the brain and behavior. This is the first study to report the phenotype of protein malnutrition and EAA supplementation in aged mice.

In this study, LPD mice showed a significantly decreased passive avoidance response compared to NPD mice, indicating that LPD in aged mice was associated with learning and memory impairment. The PAT is one of the most widely used tests for fear learning and memory. Previous studies have demonstrated that several mouse models of AD, such as rTg2576, APP23, APP/PS, and 3xrTg, show impaired learning and memory function evaluated by the PAT (13–15), similar to our data on aged mice fed a LPD. Furthermore, LPD mice showed an increase in the proportion of time spent in the open arms of the EPM, indicating that those mice had agitation and disinhibition potentially caused by the LPD. Furthermore, similar to rTg2576 mice (16–18), LPD mice showed increased total distance moved, indicating hyperactive behavior in a new environment.

Interestingly, LPD mice showed decreased amino acid concentrations in the blood and brain. Since EAAs in the blood enter the brain via the blood-brain barrier (BBB), both blood and brain EAAs can conceivably be influenced by food intake. Most neurotransmitters are synthesized from amino acids. For example, dopamine and norepinephrine are synthesized from tyrosine, which is a metabolite of Phe. Glu is synthesized from branched-chain amino acids or glutamine (Gln), which are derived from the blood via the BBB. Despite the slow flux of Leu into the brain, which is 14.5 times slower than that of Gln, 30–50% of the amino groups of Glu and Gln are derived from Leu (19). With age, the synthesis of these neurotransmitters is known to decline in humans and mice (20–23). Furthermore, the amount of neurotransmitters, including dopamine, norepinephrine, acetylcholine, Glu, serotonin and GABA, and the levels of their synthetic enzymes are known to be lower in patients with AD than in healthy people (21, 24). Dopamine and norepinephrine are monoamines that are associated with cognitive function, particularly working memory (25). In this study, LPD mice exhibited decreased concentrations of GABA, Glu, Gly, dopamine, norepinephrine, serotonin and Asp, which might be associated with behavioral abnormalities.

In this study, we used seven essential amino acids (Val, Leu, Ile, Lys, Phe, His, and Trp) that can be a source of neurotransmitters in the brain to make two EAA mixtures of different compositions. We hypothesized that the rate of amino acid influx to the brain (12) would be important and set C1 as the composition that is the reciprocal of what easily passes through the brain. In contrast, C2 was composed to directly match the ratios of the brain influx rate of the different EAAs. Although both C1 and C2 reversed the behavioral changes in the EPM, only C2 reversed the behavioral change in the PAT. C2 but not C1 improved the LPD-induced learning and memory behavior deficits and elevated the Glu concentration. The C2 mix is mainly composed of Leu, Phe, and Lys, which are potential substrates for synthesizing Glu in brain cells (19). Glu is known to be an important neurotransmitter that triggers *de novo* spine growth (26) and is involved in learning and memory ability (27). Glu restoration could be one of the key mechanisms connecting behavior and nutrition. The details of the link between the decreased amino acid and neurotransmitter concentrations and behavioral abnormalities must be further examined in the future. Also, in this study, only male mice were fed a LPD for 2 months. The effects with shorter- and longer-term LPD feeding to behaviors are to be investigated in the future. And whether the similar results will be obtained in female mice, which have estrus cycle that affect animal behaviors including emotion-related behaviors, social behaviors, and cognition, would be a future research question.

Several reports have indicated that the amount of protein consumed by the elderly is not sufficient (28–31). Oral issues such as decreased appetite with age (32, 33), dysphagia (34), reduced muscle strength required for meat consumption (35, 36), and periodontal disease (37) are noted as causes. In addition, aging of the digestive organs and gastric acid secretion decrease in the elderly (38), suggesting a decrease in digestive function to efficiently absorb the ingested protein. Although some nutritional epidemiological studies suggest the relationships between dietary protein deficiency and cognitive decline (7–10), it is yet to be demonstrated whether the EAA supplementation could affect cognitive ability in humans. Future clinical trials to examine the effects of EAA supplemental intake to cognitive ability in the elderly are needed. This study may shed light on the roles of EAAs in relation to the brain function of aged people. Although further research is necessary to illustrate the detailed mechanism and clinical effectiveness, EAA ingestion could be one possible solution for maintaining healthy brain function.

## Conclusion

In this work, we investigated the association between protein intake and cognitive function in aged mice, showing that LPD resulted in learning disabilities, disinhibition, and hyperactive behavior. LPD intake may conceivably cause low blood amino acid levels, resulting in neurotransmitter deficiency in the brain. The addition of seven EAAs (Val, Leu, Ile, Lys, Phe, His, and Trp) that can be a source of neurotransmitters to the LPD reversed some of the changes in behavior and neurotransmitter concentrations. Further studies elucidating the connection between brain function and protein and amino acid nutrition are necessary.

## Data Availability Statement

All datasets generated for this study are included in the article/Supplementary Material, and by request to the corresponding author.

## Ethics Statement

The animal study was reviewed and approved by Animal Study Ethics Committe of Ajinomoto Co., Inc.

## Author Contributions

HS, MT-Y, YT, KS, KN, MH, and AK: concept and design of the study. HS, MT-Y, NK, KM, SU, MK, MN, SK, and MI: data acquisition and analysis. HS, YT, KN, and AK: drafting the manuscript and figure. All authors read and approved the final version of the manuscript.

### Conflict of Interest

HS, MT-Y, NK, KM, SU, MK, MN, SK, MI, KS, KN, AK are employed by Ajinomoto Co., Inc. The remaining authors conducted research funded by Ajinomoto Co., Inc.
