# Supplementary material for: Protein Deficiency-Induced Behavioral Abnormalities and Neurotransmitter Loss in Aged Mice Are Ameliorated by Essential Amino Acids
Source: Front Nutr. 2020 Mar 11;7:23. doi: 10.3389/fnut.2020.00023 (PMC7079559; doi:10.3389/fnut.2020.00023)
Supplement: Supplementary file 1 [file Table_1.DOCX]

**Supplementary Table 1**

Composition of experimental diets

|  | NPD (%) | LPD (%) |
| --- | --- | --- |
| Casein | 20.00 | 5.00 |
| Cornstarch | 39.75 | 51.01 |
| α-Starch | 13.20 | 16.94 |
| Sucrose | 10.00 | 10.00 |
| Soybean oil | 7.00 | 7.00 |
| Cellulose | 5.00 | 5.00 |
| Vitamin mix (AIN93) | 1.00 | 1.00 |
| Mineral mix(AIN93G) | 3.50 | 3.50 |
| Cystine | 0.30 | 0.30 |
| Choline bitartrate | 0.25 | 0.25 |
| TBHQ | 0.0014 | 0.0014 |
| Total | 100 | 100 |

TBHQ, tertiary butylhydroquinone.
