# Supplementary material for: Protein Deficiency-Induced Behavioral Abnormalities and Neurotransmitter Loss in Aged Mice Are Ameliorated by Essential Amino Acids
Source: Front Nutr. 2020 Mar 11;7:23. doi: 10.3389/fnut.2020.00023 (PMC7079559; doi:10.3389/fnut.2020.00023)
Supplement: Supplementary file 2 [file Table_2.docx]

**Supplementary Table 2**

Plasma-free amino acid profiles of the normal protein diet group (NPD) and low protein diet group (LPD).

| Amino acids | Concentrations (μmol/L, mean ± SEM) | | p-value |
| --- | --- | --- | --- |
|  | NPD (n=12) | LPD (n=12) |  |
| Alanine | 698.3 ± 85.9 | 826.5 ± 52.0 | 0.226 |
| Arginine | 81.0 ± 5.7 | 86.6 ± 5.7 | 0.510 |
| Asparagine | 77.2 ± 14.8 | 53.0 ± 3.7 | 0.142 |
| Aspartate | 11.7 ± 2.8 | 8.3 ± 0.5 | 0.267 |
| Citrulline | 48.0 ± 2.5 | 53.6 ± 3.4 | 0.193 |
| Glutamine | 627.7 ± 48.0 | 738.5 ± 23.8 | 0.057 |
| Glutamate | 41.1 ± 5.7 | 41.5 ± 3.1 | 0.945 |
| Glycine | 199.3 ± 17.1 | 205.7 ± 19.0 | 0.802 |
| Histidine | 85.3 ± 9.0 | 71.5 ± 5.0 | 0.207 |
| Isoleucine | 133.3 ± 16.9 | 54.2 ± 4.1 | < 0.001*** |
| Leucine | 210.5 ± 27.7 | 93.1 ± 7.3 | < 0.001*** |
| Lysine | 408.3 ± 39.6 | 294.8 ± 21.2 | 0.023* |
| Methionine | 84.8 ± 10.1 | 46.2 ± 2.3 | 0.002** |
| Phenylalanine | 78.8 ± 11.4 | 41.6 ± 2.7 | < 0.001*** |
| Proline | 244.2 ± 43.8 | 144.3 ± 11.1 | 0.046* |
| Serine | 180.7 ± 24.6 | 161.9 ± 8.2 | 0.492 |
| Taurine | 451.2 ± 28.6 | 456.2 ± 86.9 | 0.955 |
| Threonine | 273.3 ± 25.2 | 170.5 ± 9.8 | 0.001** |
| Tryptophan | 90.1 ± 6.9 | 63.8 ± 3.9 | 0.004** |
| Tyrosine | 129.2 ± 21.2 | 72.2 ± 5.2 | 0.022* |
| Valine | 349.5 ± 36.2 | 139.3 ± 7.3 | < 0.001*** |

*p < 0.05, **p < 0.01, ***p < 0.001
