# Supplementary material for: Protein Deficiency-Induced Behavioral Abnormalities and Neurotransmitter Loss in Aged Mice Are Ameliorated by Essential Amino Acids
Source: Front Nutr. 2020 Mar 11;7:23. doi: 10.3389/fnut.2020.00023 (PMC7079559; doi:10.3389/fnut.2020.00023)
Supplement: Supplementary file 3 [file Table_3.docx]

**Supplementary Table 3**

The concentrations of free amino acids in the prefrontal cortex area of the brain in the normal protein diet group (NPD) and low protein diet group (LPD).

| Amino acids | Concentrations (pmol/mg tissue, mean ± SEM) | | p-value |
| --- | --- | --- | --- |
|  | NPD (n=12) | LPD (n=12) |  |
| Alanine | 82.7 ± 3.4 | 82.4 ± 2.6 | 0.951 |
| Arginine | 8.8 ± 0.3 | 10.2 ± 0.7 | 0.087 |
| Asparagine | 9.5 ± 0.3 | 8.2 ± 0.3 | 0.012* |
| Aspartate | 275.5 ± 9.2 | 245.7 ± 5.0 | 0.012* |
| GABA | 185.9 ± 5.0 | 166.9 ± 4.5 | 0.011* |
| Glutamine | 566.7 ± 48.2 | 479.0 ±13.4 | 0.107 |
| Glutamate | 1295.5 ± 35.4 | 1156.0 ± 16.3 | 0.002** |
| Glycine | 88.3 ± 2.6 | 75.6 ± 2.7 | 0.003** |
| Histidine | 8.4 ± 0.8 | 8.9 ± 0.4 | 0.654 |
| Isoleucine | 3.2 ± 0.1 | 2.3 ± 0.1 | < 0.001*** |
| Leucine | 6.4 ± 0.4 | 4.7 ± 0.2 | < 0.001*** |
| Lysine | 14.8 ± 0.5 | 12.9 ± 0.4 | 0.009** |
| Methionine | 4.2 ± 0.3 | 3.9 ± 0.1 | 0.369 |
| Phenylalanine | 4.8 ± 0.5 | 4.0 ± 0.1 | 0.166 |
| Proline | 8.6 ± 0.3 | 7.1 ± 0.2 | < 0.001*** |
| Serine | 72.2 ± 2.1 | 63.7 ± 1.5 | 0.004** |
| Taurine | 788.4 ± 23.8 | 701.2 ± 13.3 | 0.005** |
| Threonine | 29.7 ± 1.0 | 22.9 ± 1.2 | < 0.001*** |
| Tryptophan | 1.5 ± 0.1 | 1.4 ± 0.1 | 0.423 |
| Tyrosine | 8.2 ± 1.0 | 7.6 ± 0.2 | 0.521 |
| Valine | 8.9 ± 0.4 | 6.0 ± 0.2 | < 0.001*** |

*p < 0.05, **p < 0.01, ***p < 0.001
