# Supplementary figures and images for: Protein Deficiency-Induced Behavioral Abnormalities and Neurotransmitter Loss in Aged Mice Are Ameliorated by Essential Amino Acids
Source: Front Nutr. 2020 Mar 11;7:23. doi: 10.3389/fnut.2020.00023 (PMC7079559; doi:10.3389/fnut.2020.00023)

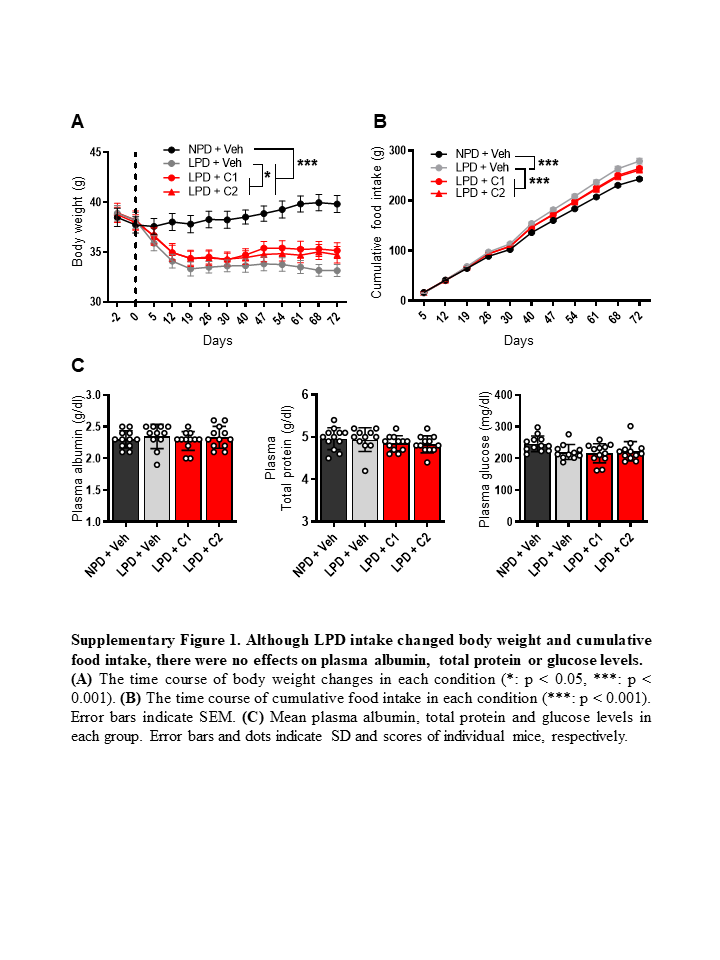

Supplement: Supplementary file 4 [file Image_1.TIF]
